# Preventive role of green tea catechins from obesity and related disorders especially hypercholesterolemia and hyperglycemia

**DOI:** 10.1186/s12967-015-0436-x

**Published:** 2015-03-04

**Authors:** Rabia Shabir Ahmad, Masood Sadiq Butt, M Tauseef Sultan, Zarina Mushtaq, Shakeel Ahmad, Saikat Dewanjee, Vincenzo De Feo, Muhammad Zia-Ul-Haq

**Affiliations:** Department of Food Science, Nutrition and Home Economics, Govt College University Faisalabad, Faisalabad, Pakistan; National institute of Food Science and Technology, University of Agriculture Faisalabad, Faisalabad, Pakistan; Department of Food Science and Technology, Bahauddin Zakariya University, Multan, Pakistan; Department of Agronomy, Bahauddin Zakariya University, Multan, Pakistan; Advanced Pharmacognosy Research Laboratory, Department of Pharmaceutical Technology, Jadavpur University, Kolkata, 700032 India; Department of Pharmacy, University of Salerno, Fisciano, Salerno, 84084 Italy; The Patent Office, Karachi, Pakistan

**Keywords:** Green tea, Functional drink, Catechins, EGCG, Hypercholesterolemia, Hyperglycemia

## Abstract

**Background:**

During the last few years, scientific investigations have proposed diet based regimens to prevent several health ailments including obesity, hypercholesterolemia and diabetes. In this regard, a promising tool is the use of functional foods/nutraceuticals. Present research project was an attempt to explore nutraceutical worth of locally grown green tea variety (Qi-Men) against lifestyle related disorders.

**Methods:**

Functional drinks (T_2_ and T_3_) were prepared by adding catechins and epigallocatechin gallate (EGCG) @ 550 mg/500 mL and compared with control (T_1_). These functional drinks were tested in experimental rats modeling (Sprague Dawley). Based on diets, four studies were conducted i.e. trial-I (normal diet), trial-II (high cholesterol diet), trial-III (high sucrose diet), trial-IV (high cholesterol + high sucrose diet). Rats were monitored daily for their feed and drink intake while body weight was measured on weekly basis. After period of 56 days rats were sacrificed and evaluated their serum lipid (cholesterol, LDL and HDL), glucose and insulin levels.

**Results:**

Results for feed consumption by rats revealed that highest feed intake was recorded in group provided control drink than other groups. However, non significant differences were noted among all groups for drink consumption. Functional drinks resulted in significant reduction in body weight with maximum lowering noted in trial-II and III i.e. 10.73 to 8.49% and 10.12 to 10.49%, respectively. Likewise, cholesterol and LDL were substantially reduced with 14.42% decrease observed in trial-IV and 30.43% in trial-II, respectively. Furthermore, serum glucose and insulin levels were also lowered significantly in the trial-III and IV while in trial-I and II differences were non-significant. In contrast to lipid profile, experimental drink containing EGCG reduced the trait better than catechins based functional drink.

**Conclusions:**

The drinks supplemented with catechins and EGCG are effective against obesity, hypercholesterolemia and hyperglycemia.

## Background

Changing lifestyle and poor dietary habits of people often lead to development of various maladies like obesity, diabetes, dyslipidemia and immune dysfunction. Obesity is now well-known as one of the global concerns because it is not only prevalent in the developed nations but also becoming common in the developing world with associated physiological threats. Dyslipidemia is more frequent in obese people with raised levels of LDL, triglycerides and cholesterol. Among contributory factors of obesity, dietary habits are considered one of reasons for its expansion. During the last few years, amplified consumption of carbohydrate and animal fat has contributed to increased incidence of atherosclerosis induced cardiovascular complications and diabetes mellitus. High cholesterol diet causes significant increase in serum cholesterol level along with sensitivity to oxidation. Sucrose rich diet induces hyper-triglyceridemia and hypercholesterolemia [1].

Phytochemicals are endemic in the human diet from the ancient times to fight against diseases as most of the medicines have been derived from plants. In the recent era, diet based therapy has been revitalized globally and people are adopting the approach of using natural materials as an intervention against various ailments. During the last few years, scientific investigations have proposed several modules like diet based regimen to prevent life threatening disorders including obesity, hypercholesterolemia and diabetes. Among these strategies, a promising tool is the use of functional/nutraceuticals foods that not only improve consumer health and wellness but also reduce disease risk with minimal cost. Green tea (*Camellia sinensis*) belonging to family *Theaceae* is one such example of functional drinks containing bioactive molecules holding cure against various diseases. Green tea contains appreciable amounts of phytochemicals especially catechins [2]. According to literature, dried tea leaves mainly contain 10–25% polyphenols mainly belonging to various classes i.e. flavonols and, flavonoids, and flavondiols. Green tea catechins are further comprised of different chemical moieties that inlcue epigallocatechin-3-gallate (EGCG), epicatechin (EC), epicatechin-3-gallate (ECG), epigallocatechin (EGC). Amongst these, EGCG is present in higher amounts and considered to be an effective antioxidant with appreciable radical scavenging abilities [3].

Owing to the prophylactic role of green tea, its consumption is escalating in the vulnerable segments to cope with health disorders thus besides being a traditional beverage acts as an intervention against various physiological threats. Keeping in view the lifestyle disorders, present project was designed to use locally grown green tea variety Qi-Men with special reference to its active ingredient namely catechins. Concurrently, functional drinks containing the health ingredients i.e. catechins and EGCG were the limelight of the present research investigation. The therapeutic role of functional drinks was explored against lifestyle disorders.

## Methods

### Functional drink preparation

Functional drinks (T_2_ and T_3_) were prepared by addition of catechins and epigallocatechin gallate (EGCG) respectively. A control drink, T_1_ (without active ingredient) was also prepared for comparison purpose. Other ingredients included aspartame, citric acid, sodium benzoate, carboxy methyl cellulose (CMC), food grade color and flavor. After processing of drinks, active ingredients including catechins and EGCG were added individually @ 550 mg/500 mL in respective drinks. Briefly, catechins rich fraction from green tea was separated using water at constant temperature of 40°C and concentrated through a rotary evaporator (Eyela, Japan) and dried through Vacuum Oven. Later, chloroform and ethyl acetate were used used to extract catechins from dried extracts. For extracting EGCG, water filtration followed by addition of caffeine and subsequent centrifugation at 16,600×g for 20 min was used. Later, pellet was decaffeinated and solvents (chloroform and ethyl hexanoate) fractionation was used for further separation of gallate rich moieties. The resultant aqueous fraction thus obtained was subjected to further partition with propyl acetate to get EGCG rich fraction that was further concentrated using a Rotary Evaporator and dried using Freeze Drier [4].

### Test animals and their housing

The research received ethical approval from Animal Care Committee (ACC) of National Institute of Food Science and Technology (NIFSAT). Later, National Institute of Health (NIH) provided the researchers with 120 infectious free Sprague dawley rats for the research purpose. The rats were housed in the Animal Room of National Institute of Food Science and Technology. Four different trials depending on different types of diets i.e. trial-I (normal diet), trial-II (high cholesterol diet), trial-III (high sucrose diet) and trial-IV (high cholesterol + high sucrose diet) were conducted. Therfore, 120 rats were divided in four groups of thirty each. In each trial, rats were further divided into three subgroups comprising of ten rats in each subgroup. Rats were placed in standard polypropylene cages with stainless steel top grill. Rats were acclimatized by feeding basal diet (AIN-76A) for a period of one week. At the initiation of study some rats were sacrificed to get baseline values. The temperature (23 ± 2°C) and relative humidity (55 ± 5%) were maintained throughout the experiment with 12 hours light–dark period. Feed & drink intake was recorded daily whilst body weight on weekly basis throughout the experiment. Spilled diet and feces were also collected.

### Trial-I: normal diet

In trial-I normal diet was provided to rats. The normal diet contained corn oil (10%), casein protein (10%), corn starch (66%), casein (10%) and cellulose (10%). The mineral and vitamin mixtures (AIN-76) were added @ 3% and 1%, respectively. The functional drinks (T_1_, T_2_, T_3_) were provided in graduated bottles for a period of 8 weeks (Table 1). Following similar approach, three other studies were conducted to determine the impact of functional drinks against respective diets.

**Table 1:** **Diet plan used in the studies**

	**(Trial-I)**			**(Trial-II)**			**(Trial-III)**			**(Trial-IV)**		
	**Normal diet**			**High cholesterol diet**			**High sucrose diet**			**High cholesterol + high sucrose diet**		
Groups	1	2	3	1	2	3	1	2	3	1	2	3
Drinks	T_1_	T_2_	T_3_	T_1_	T_2_	T_3_	T_1_	T_2_	T_3_	T_1_	T_2_	T_3_

T_1_: Control.

T_2_: Functional drink containing catechins.

T_3_: Functional drink containing EGCG.

### Trial-II: high cholesterol diet

In trial-II, high cholesterol diet containing 1% of cholesterol was distributed to the normal rats along with other ingredients provided in normal diet. The functional drinks were also provided to the rats groups simultaneously to synchronize their effect on the respective category.

### Trial-III: high sucrose diet

In trial-III, high sucrose diet containing 40% sucrose was added to corn oil (10%), corn starch (26%), casein (10%), cellulose (10%) and salt & vitamin mixture (3 & 1%).

### Trial-IV: high cholesterol + high sucrose diet

Rats of group IV were fed on diet including corn oil (10%), corn starch (25%), casein (10%), cellulose (10%), salt & vitamin mixture (3 & 1%), cholesterol and sucrose in amount of 1% and 40% respectively.

The following parameters were recorded separately in all rat modeling studies.

### Feed and drink intake

Net feed intake of each group was measured daily by excluding spilled diet from the total diet during the entire study period [5]. The functional drink intake of each group was also recorded daily by monitoring the differences in the graduated bottles.

### Body weight gain

Gain in body weight of experimental groups was measured weekly throughout the study period to monitor suppressing effect of functional drinks on body weight gain.

### Serum lipid profile

Serum cholesterol level was determined using CHOD–PAP method [6]. High density lipoprotein (HDL) in serum samples was measured by HDL Cholesterol Precipitant method and low density lipoproteins (LDL) following the procedure of McNamara *et al*. [7]. Total triglycerides were determined by liquid triglycerides (GPO–PAP) method [8].

### Serum glucose and insulin levels

In each study, rats serum samples were evaluated for glucose concentration by GOD-PAP method as described by Thomas and Labor [9] whereas insulin level was assessed following the method of Besch [10].

### Statistical analysis

Completely Randomized Design (CRD) was applied and resultant data was subjected to statistical analysis using Cohort version 6.1. Analysis of variance technique (ANOVA) was used to determine the level of significance and means were further compared through Duncan Multiple Range test.

## Results

### Feed and drink intake

**Figure 1:**
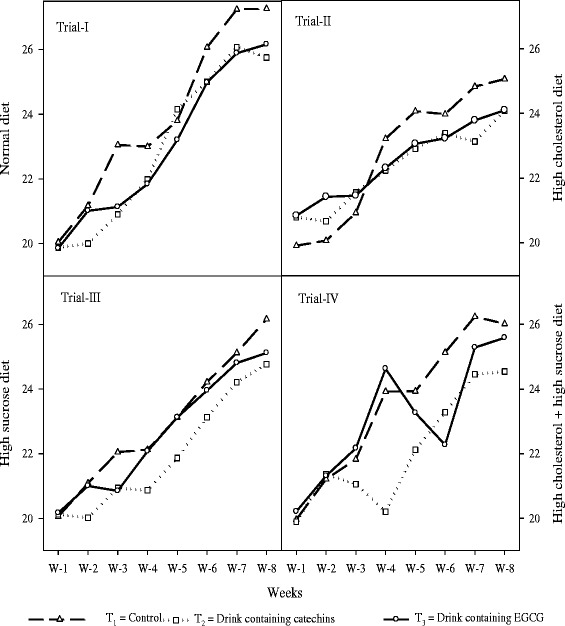
**Feed intake (g/rat/day) to show the effects of functional drinks in different studies fed with diets i.e. normal, high sucrose, high cholesterol, and high sucrose + high cholesterol.** The results were clearly indicating the differences in different trial, however, within each trial, feed intake varied differently. Normally, feed intake in all the experimental groups remain similar in the first four weeks and later varied significantly.

Means regarding feed intake (Figure 1) in all trials (I, II, III and IV) indicated that maximum feed intake was recorded in T_1_ group (control drink). Whereas, comparatively, lower feed intakes were noted in T2 (catechins based functional drink) and T_3_ (EGCG based functional drink) groups. In all studies, there was a progressive increase in feed intake with passage of time however; relatively lower feed intake was recorded in groups using experimental functional drinks with green tea active ingredients.

**Figure 2:**
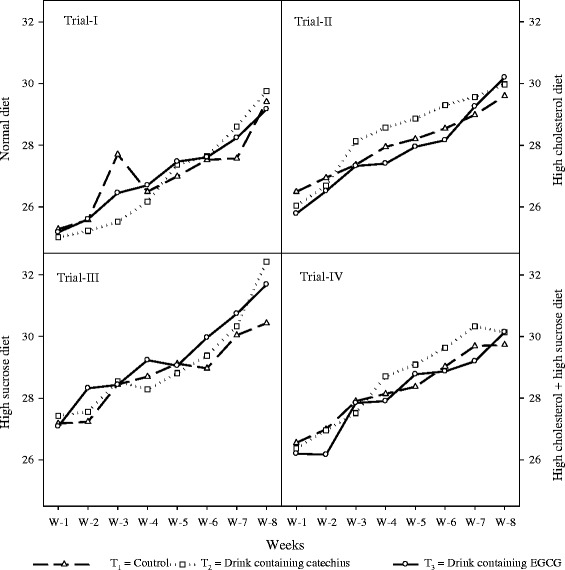
**Drink intake (mL/rat/day) to show the effects of functional drinks in different studies fed with diets i.e. normal, high sucrose, high cholesterol, and high sucrose + high cholesterol.** The results showed slight but non-significant variations. It clearly indicates that functional drinks containing catechins and EGCG remained at par with normal drink.

Means belonging to drink intake of rats (Figure 2) in four studies revealed that there was an increase in drink consumption during the entire trial with non-significant differences among the treatments thus showing suitability of the product.

### Body weights

**Figure 3:**
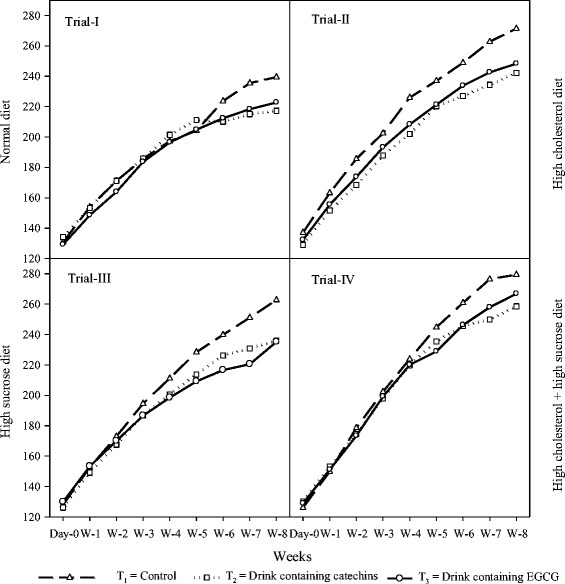
**Body weight in different studies (g/rat) to show the effects of functional drinks in different studies fed with diets i.e. normal, high sucrose, high cholesterol, and high sucrose + high cholesterol.** The results showed significant variations in different trial. However, the effects are directly linked with feed intake as higher feed intake results in higher body weight and vice versa.

Body weights of different groups of rats in all studies are shown in Figure 3 showing that more weight gain was recorded in rats groups consuming control drink (T_1_) than functional drinks (T_2_ and T_3_). The trend for body weights showed differences in each trial e.g. higher body weights were recorded in high cholesterol fed diets. Moreover, body weights in all groups increased steadily in the first 28 days (4 weeks) but last four weeks provided insight about the differences in body weights. In all the trials, body weight of rats in experimental groups was less than placebo or control. The effects of catechins based functional drinks were more pronounced in high cholesterol diets, while EGCG based functional drink performed better in high sucrose diet trial and high cholesterol + high sucrose diet trial.

### Lipid profile

Table 2 showed that functional drink consumption affected cholesterol significantly in all studies. However drink T_2_ (drink containing catechins) was more effective in reducing cholesterol than T_3_ (drink containing EGCG) though the differences were non-significant. It is obvious from Table 2 that drinks exhibited non-significant differences on HDL level, while LDL level in different groups of rats was significantly affected by functional drinks in all studies. The values showed non-significant effects of drinks on triglycerides in trial-I and II but significant differences were recorded for trial-III and IV. Total cholesterol varied from 74.73 ± 2.34 to 78.64 ± 3.07 mg/dL in normal rats, while the cholesterol level was higher in all other studies. Moreover, it can be observed from the results that functional drink containing catechins was more effective in reducing the cholesterol level. However, the percentage reduction was higher in group of rats fed on high cholesterol diet, and high cholesterol + high sucrose diets. The trend for high density lipoproteins was opposite to total cholesterol. However, the increasing tendency as a function of functional drink was statistically non-significant. The results regarding low density lipoproteins (LD) indicated that it varied from 25.55 ± 1.22 to 30.34 ± 1.43 mg/dL in normal rats, while it was significantly higher in all studies. The functional drink containing green tea catechins decreased the trait significantly. However, the reduction was higher in group of rats fed on high cholesterol diet (30.44%), and high cholesterol + high sucrose diets (28.81%) in the same experimental group. In contrast to catechins based functional drink, the EGCG based functional decreased LDL significantly but its effect was slightly less as compared to catechins supplemented group. The triglycerides levels expressed in mg/dL also varied as a functional of experimental diets as mentioned earlier. The level for same trait was lower in normal rats but significantly higher in groups of rats fed on cholesterol and sucrose or their combination. The functional drinks decreased the trait significantly. Functional drinks containing catechins and EGCG reduced triglycerides by 6.68 and 4.42%, respectively, in rats fed on high cholesterol diet. Similarly, the same experimental diets reduced the trait by 11.18 and 3.23%, respectively, in high sucrose fed rats. However, booth drinks performed equally better in groups of rats fed high cholesterol and high sucrose diet together i.e. 12.49 and 10.89% reduction in triglycerides, respectively.

**Table 2:** **Effect of functional drinks on lipid profile in rats**

	**Studies**	**Treatments**		
		**T**_**1**_	**T**_**2**_	**T**_**3**_
**Cholesterol (mg/dL)**	**Trial-I**	78.64 ± 3.07a	74.73 ± 2.34b	76.46 ± 2.42ab
	**Trial-II**	147.02 ± 7.83a	126.42 ± 4.47b	130.09 ± 7.77b
	**Trial-III**	128.74 ± 5.60a	113.84 ± 6.93b	116.79 ± 5.36b
	**Trial-IV**	155.02 ± 8.36a	132.67 ± 6.90b	135.42 ± 6.33b
**HDL (mg/dL)**	**Trial-I**	34.64 ± 2.57	35.80 ± 1.79	35.47 ± 2.25
	**Trial-II**	56.29 ± 3.71	58.30 ± 4.47	57.80 ± 4.15
	**Trial-III**	46.95 ± 2.59	48.27 ± 3.53	47.80 ± 3.43
	**Trial-IV**	58.86 ± 3.01	60.35 ± 4.66	59.27 ± 2.61
**LDL (mg/dL)**	**Trial-I**	30.34 ± 1.43a	25.55 ± 1.22b	27.41 ± 1.46ab
	**Trial-II**	69.68 ± 3.45a	48.47 ± 2.41b	52.17 ± 3.79b
	**Trial-III**	64.66 ± 3.22a	50.36 ± 2.83b	52.42 ± 4.32b
	**Trial-IV**	74.59 ± 4.95a	53.10 ± 3.45b	57.27 ± 4.98b
**Triglycerides (mg/dL)**	**Trial-I**	68.30 ± 3.74	66.92 ± 3.61	67.89 ± 3.13
	**Trial-II**	105.26 ± 6.02	98.23 ± 5.16	100.61 ± 7.71
	**Trial-III**	85.63 ± 6.52a	76.06 ± 5.09b	82.86 ± 4.82ab
	**Trial-IV**	107.85 ± 7.21a	94.38 ± 6.05b	96.10 ± 4.69b

Values are expressed as means ± SD.

Means carrying same letter in a row do not differ significantly.

Means showing different lettering in a row differed significantly at P < 0.05.

Trial-I: Normal diet.

Trial-II: High cholesterol diet.

Trial-III: High sucrose diet.

Trial-IV: High cholesterol + high sucrose diet.

T_1_: Control drink (without active ingredients).

T_2_: Drink containing catechins.

T_3_: Drink containing EGCG.

### Glucose and insulin level

Table 3 also reveals that that treatments exhibited significant differences on glucose and insulin in the trial-III and IV while in trial-I and II differences were non-significant. The results indicated that glucose level varied from 90.44 ± 4.79 to 93.55 ± 6.01 mg.dL in normal rats, while in high cholesterol fed rats, the same trait varied from 101.51 ± 4.58 to 105.60 ± 6.61 mg/d. The results showed some significant glucose lowering abilities of functional drink in group of rats fed on high sucrose or high cholesterol and sucrose diets. In contrast to lipid profile, experimental drink containing EGCG reduced the trait better than catechins based functional drink. Although, variations between functional drinks were statistically non-significant but higher decrease in glucose levels was more important. The trait decreased by 10.10 and 12.33% in catechins and EGCG based functional drinks, respectively. The glucose level decreased by 6.91 and 8.06% in experimental diets fed on both (cholesterol and sucrose), respectively. The results regarding insulin showed direct proportionality with glucose. The decrease in glucose also witnessed slight decreased amounts of insulin but the decrease was more prominent in control groups. Although, statistically analysis revealed significant variations in insulin but insulin secretions are not influenced quite significantly according to researchers point of view. Glucose lowering properties of functional drinks containing catechins and EGCG could not correlate with insulin but the effects could be due to their antioxidant nature.

**Table 3:** **Effect of functional drinks on glucose and insulin levels of rats**

	**Studies**	**Treatments**		
		**T**_**1**_	**T**_**2**_	**T**_**3**_
**Glucose (mg/dL)**	**Trial-I**	93.55 ± 6.01	92.44 ± 4.43	90.44 ± 4.79
	**Trial-II**	105.60 ± 6.61	103.32 ± 5.26	101.51 ± 4.58
	**Trial-III**	125.93 ± 5.47a	113.21 ± 7.25b	110.40 ± 5.85b
	**Trial-IV**	130.05 ± 8.75a	121.07 ± 7.32b	119.57 ± 6.60b
**Insulin (μU/mL)**	**Trial-I**	9.33 ± 0.51	9.15 ± 0.68	9.14 ± 0.71
	**Trial-II**	11.05 ± 0.09	10.59 ± 0.15	10.34 ± 0.65
	**Trial-III**	14.34 ± 0.41a	12.65 ± 0.57b	12.20 ± 0.49b
	**Trial-IV**	16.34 ± 0.59a	14.20 ± 0.74b	13.65 ± 0.67b

Values are expressed as means ± SD.

Means carrying same letter in a row do not differ significantly.

Means showing different lettering in a row differed significantly at P < 0.05.

Trial-I: Normal diet.

Trial-II: High cholesterol diet.

Trial-III: High sucrose diet.

Trial-IV: High cholesterol + high sucrose diet.

T_1_: Control drink (without active ingredients).

T_2_: Drink containing catechins.

T_3_: Drink containing EGCG.

## Discussion

In present exploration, less feed consumption due to functional drinks is supported by the work of Babu *et al.* [11]. Results for drink consumption are similar to work of Yang *et al*. [12]. However, Lee *et al*. [13] elucidated suppressed fluid intake in a group provided EGCG solution. The differences may be due to the fact that they used EGCG solution without toting up any additive. However, in the present case drinks were prepared by adding flavor and artificial sweetener that may be a reason for improved consumption.

The current results showing reduced body weight gain in groups consuming drinks containing catechins or EGCG are in consistent with the study of Basu *et al.* [14] and Byun *et al.* [15] Weight reduction by green tea might be due to reduced digestibility and an increase in energy expenditure and fat oxidation through β-adrenoceptor activated thermogenesis of brown adipose tissue [16,17]. Inhibited lipid absorption from meals might be other reason for reduced weight gain [18].

Results for decreased cholesterol and LDL levels are in consistent with the work of Kim *et al.* [19]. Hypocholesterolemic potential of catechins can be accredited to increased fecal excretion of cholesterol and bile acid [20]. Tea catechins increase the bile acid excretion by preventing reabsorption from small intestine through disruption of micelle formation of bile acid. This increased excretion of bile acid and cholesterol activates cholesterol 7α-hydroxylase that enhances the conversion of liver cholesterol to bile acid to restock this loss thus resulting in cholesterol reduction. This decrease in hepatic cholesterol content in turn stimulates LDL receptor expression and lowers blood cholesterol level [21].

Results for non-significant effect of drinks on HDL are in agreement with previous work of Gomikawa *et al*. [22]. The effects could be due to presence of various bioactive components present in green tea [4]. Roghani and Baluchnejadmojarad [23] noted LDL reduction in diabetic rat modeling with EGCG. The proposed mechanism of LDL reduction by green tea catechins is through inhibition of cholesterol synthesis and dietary cholesterol absorption [24]. Crude catechins decrease plasma cholesterol concentrations by upregulating LDL receptor. The increase in the LDL receptor improves the uptake of low density lipoprotein cholesterol from the blood circulation [25]. Moreover, reduced expression of 3-hydroxy-3-methylglutaryl coenzyme A reductase (HMGR) might be another reason for hypocholesterolemic activity of green tea as green tea EGCG also inhibits HMGR activity [26,27].

Low concentration of triglycerides by green tea might result from suppressed expression of stearoyl-CoA desaturase (SCD 1) gene. Because in liver triglyceride synthesis depends on the expression of the SCD 1 gene, that is involved in biosynthesis of oleate and palmitoleate, the major monounsaturated fatty acids of triglycerides. However, Watanabe *et al*. [28] is of the view that reduced activity of acetyl-Co A by green tea catechins is a reason for low triglycerides synthesis.

Reduced glucose level by green tea is supported by the work of Polychronopoulos *et al*. [29] that stated that there occurs inverse relation between green tea and blood glucose. The antihyperglycemic effect of green tea may be due to activated uptake of glucose, inhibited intestinal glucose transporter and decreased expression of gluconeogenesis controlling genes [30]. EGCG exhibits hypoglycemic potential by preventing the intestinal glucose absorption via sodium-dependent glucose transporter (SGLT1), lowering the expression of mRNA for gluconeogenesis controlling enzymes [31] and causing repression of glucose production, phosphoenolpyruvate carboxykinase (PEPCK) and glucose-6-phosphatase gene expression, hepatocyte nuclear factor 1α (HNF1α), and HNF4α in cells [32]. Additionally, EGCG has direct effect on hepatic glucose metabolism thus improving glucose stimulated insulin secretion [32]. Attenuation of insulin level with functional drinks in current exploration is supported by the work of Hsu *et al.* [33]. It is proposed that amelioration of insulin resistance by green tea is associated with increased expression of glucose transporter (GLUT) IV [34]. It can be concluded that green tea mitigates hypercholesterolemia and hyperglycemia effectually. It has cholesterol lowering properties along with hypoinsulinemic capability that could be associated with improved insulin sensitivity.

## Conclusions

Our study demonstrates that drinks supplemented with catechins and EGCG are effective against obesity, hypercholesterolemia and hyperglycemia. Consumption of functional drink reduced the body weight. Functional drinks resulted in significant reduction in body weight that could be caused by a decreased feed intake. Moreover, improvement in lipid profile was observed in all trials but more pronounced in semi-diseased states i.e. trial-III and trial-II. Furthermore, serum glucose and insulin levels were also lowered significantly in the trial-III and IV. It was interesting to observe that catechins showed higher potency against hypercholesterolemia but EGCG reduced the hyperglycemia more effectively.
